# Evolution of the Human Brain Can Help Determine Pathophysiology of Neurodevelopmental Disorders

**DOI:** 10.3389/fnins.2022.871979

**Published:** 2022-04-01

**Authors:** Koichiro Irie, Miyuki Doi, Noriyoshi Usui, Shoichi Shimada

**Affiliations:** ^1^Department of Neuroscience and Cell Biology, Graduate School of Medicine, Osaka University, Suita, Japan; ^2^Center for Medical Research and Education, Graduate School of Medicine, Osaka University, Suita, Japan; ^3^United Graduate School of Child Development, Osaka University, Suita, Japan; ^4^Global Center for Medical Engineering and Informatics, Osaka University, Suita, Japan; ^5^Addiction Research Unit, Osaka Psychiatric Research Center, Osaka Psychiatric Medical Center, Osaka, Japan

**Keywords:** brain evolution, molecular evolution, comparative genomics, human brain, oligodendrocyte, neurodevelopmental disorders (NDDs)

## Abstract

The evolution of humans brought about a co-occurring evolution of the human brain, which is far larger and more complex than that of many other organisms. The brain has evolved characteristically in humans in many respects, including macro-and micro-anatomical changes in the brain structure, changes in gene expression, and cell populations and ratios. These characteristics are essential for the execution of higher functions, such as sociality, language, and cognition, which express humanity, and are thought to have been acquired over evolutionary time. However, with the acquisition of higher functions also comes the risk of the disease in which they fail. This review focuses on human brain evolution and neurodevelopmental disorders (NDDs) and discusses brain development, molecular evolution, and human brain evolution. Discussing the potential for the development and pathophysiology of NDDs acquired by human brain evolution will provide insights into the acquisition and breakdown of higher functions from a new perspective.

## Introduction

The human brain is relatively large, compared with those of other organisms, and it is more complex, harboring a greater capacity for higher functions such as cognition or sociality. A better understanding of the evolution of the human brain and of the pathophysiology of diseases that disrupt cognition might also improve our understanding of the mechanism of acquisition of these higher order functions. Cognitive disorders, such as neurodevelopmental disorders (NDDs) or psychiatric disorders, are thought to be human-specific disorders due to the disruption of higher cognitive functions of the brain, which most other animals lack.

The brains of humans and other primates or rodents differ in a number of neuroanatomical patterns such as wrinkles, size, morphology, and connectivity; cell biological differences such as cell ratios and population distribution; and molecular differences such as gene expression (Hill and Walsh, 2005; Sherwood et al., 2008; Defelipe, 2011; Preuss, 2011, 2017; Gabi et al., 2016; Smaers et al., 2017; Li et al., 2018; Rilling and van den Heuvel, 2018). Structurally, the enlarged human brain is unique in the layered structure of the human cerebral cortex. Unlike other primates and rodents, the structure of the ventricular zone (VZ) and subventricular zone (SVZ) is expanded by characteristic neural progenitor cells (Hill and Walsh, 2005; Lui et al., 2011; Cadwell et al., 2019). In addition, many unique characteristics of molecular evolution have been reported, such as isoforms and splicing, non-coding RNA contained in untranslated regions, RNA binding proteins (RNAbps), and amino acid substitutions (Mattick, 2004; Rajan et al., 2009; Geschwind and Konopka, 2012; Gerstberger et al., 2014; Zhu et al., 2018). The evolution of these molecules has in turn drove the evolution of a mechanism by which one gene has more than one function, thus increasing the number of cognitive functions but without significantly increasing the number of genes.

Despite the advantages of higher cognitive functions, such evolutionary brain structure and molecular complexity foster increases in various risks, such as expression and replication, in decoding genomic information and executing enormous commands. For NDDs such as autism spectrum disorder (ASD, Autism Spectrum Disorders Working Group of The Psychiatric Genomics Consortium, 2017) or attention-deficit hyperactivity disorder (ADHD), many large-scale, genome-wide association studies (GWASs) have been carried out using next-generation sequencing (NGS). The causative and related genes of these disorders have been identified, suggesting that mutations in these genes may be directly linked to the cause of NDDs (Iossifov et al., 2014; Satterstrom et al., 2020; Wang et al., 2020). Most of these causative and related genes are known to play roles in brain development and synaptogenesis (Tebbenkamp et al., 2014; Wang et al., 2014; de la Torre-Ubieta et al., 2016).

In this review, we look at the latest findings on the relationship between human brain evolution and NDDs, and explore the pathophysiology of NDDs from studies of human brain development, molecular evolution, and comparative genomics. Recently, we reported some results suggesting that the evolution of oligodendrocytes in humans accelerated the evolution of the human brain, therefore, this review also discusses the role of oligodendrocytes in brain function and NDDs.

## Neurodevelopmental Disorders

NDDs are complex disorders in which multiple genetic and/or environmental factors are involved in disease onset. Most patients with NDDs begin to show symptoms during childhood. Children with NDDs might experience difficulties with motor skills, learning and/or memory, language and/or non-verbal communication, and/or other neuropsychiatric problems. In the DSM-5, NDDs include disorders such as ASD, ADHD, intellectual disability (ID), communication disorder (CD), specific learning disorders (SLD), and motor disorders (MD).

ASD is a complex NDD characterized by impaired social communication coupled with restricted and repetitive patterns of behaviors or interests. The prevalence of ASD is approximately 1 in every 54 patients (1.85%) in the United States (Maenner et al., 2020). Genetic and environmental factors are both involved in the onset of ASD, and approximately 1,000 genes are thought to be involved (Toma et al., 2014; Griswold et al., 2015; Wilkinson et al., 2015; Yao et al., 2015; Satterstrom et al., 2020; Wang et al., 2020). ADHD is another NDD that is characterized by attention deficit, hyperactivity, and impulsivity as its main symptoms, and like ASD, it develops in childhood. ADHD affects approximately 8–12% of children worldwide (Faraone et al., 2003; Polanczyk et al., 2015). IDs are characterized by the onset of deficits in intellectual and adaptive function during the developmental period. CDs include language disorders, speech or sound disorders, and childhood-onset fluency disorders. SLDs are disorders in which children find it is difficult to read, write, and speak. Finally, MDs are characterized abnormal and involuntary movements, including developmental coordination disorders, stereotypic movement disorders, and tic disorders such as Tourette syndrome.

The commonality among all NDDs is the presence of some neurodevelopmental problem. As the brain develops, interference by genetic or environmental factors that disrupt normal development can lead to higher risk of developing NDDs. Therefore, understanding human brain evolution might contribute to a better understanding of the pathophysiology of some NDDs.

## Human Brain Evolution and Development

There are many differences between the human brain and those of other living organisms. For example, the size of synapses and number of spines in the human brain has increased over evolutionary time (Elston et al., 2001; Duan et al., 2003; Defelipe, 2011).

In human brain development, between gestational weeks (GW) four to five, neuroepithelial cells divide symmetrically at the VZ. Around GW5, neural progenitor cells, called radial glial cells, (RGs) begin to switch from symmetric to asymmetric cell division. During asymmetric division, one daughter cell remains in the VZ as a RG, while the other differentiates into either a post-mitotic neuron or an intermediate progenitor cell (IPCs) (Pontious et al., 2008). In primates, RGs and IPCs are divided into two subpopulations, apical and basal. Apical RGs and IPCs extend bipolar radial fibers between apical and basal surfaces, but basal/outer RGs (bRGs/oRGs) and basal IPCs form basal fibers. A few bRGs are present in the SVZ in rodents, whereas in gyrencephalic species such as humans, most bRGs are located in the outer SVZ (OSVZ), which is a characteristic structure of gyrencephalic species. Asymmetrically dividing bRGs in the OSVZ contribute to cortical growth and folding (Lui et al., 2011).

**FIGURE 1:**
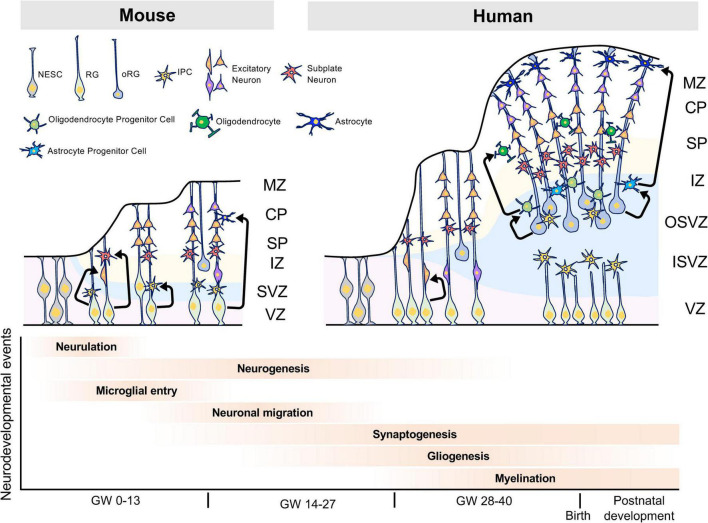
Characteristics of human brain development. The human brain has the OSVZ and ISVZ as anatomical features, while the mouse brain has only the SVZ as a thin layer. The OSVZ contains a large number of oRGs that have replication and differentiation potential, and this layer is important for the evolution and expansion of the human brain. Furthermore, the expansion of white matter by oligodendrocytes is thought to have contributed to the evolution of the human brain and the acquisition of higher brain functions. NESC, neuroepithelial stem cell; RG, radial glia cell; oRG, outer radial glia cell; IPC, intermediate progenitor cell, MZ, marginal zone; CP, cortical plate; SP, subplate; IZ, intermediate zone; SVZ, subventricular zone; VZ, ventricular zone; OSVZ, outer subventricular zone; ISVZ, inner subventricular zone; GW, gestational week.

Around GW7, post-mitotic committed neurons migrate along radial glial fibers from the VZ and SVZ to the apical brain surface and begin to form cortical plates. The cortical plate is initially formed by two layers: the marginal zone and subplate layer. The marginal zone ultimately becomes layer I, which is composed of Cajal-Retzius cells (CR-cells) and plays an important role in lamina formation *via* reelin secretion (Goffinet, 1979; Causeret et al., 2021). Human CR cells specifically express *HAR1F*, a non-coding RNA gene in human accelerated regions, which are primarily involved in human-specific cortical evolution and acquisition of higher functions (Pollard et al., 2006). The subplate layer functions for thalamocortical axon pathfinding, synapse maturation, and patterning of primary sensory areas in the cortex (Kanold and Luhmann, 2010). Recently, subplate neurons have been shown to control neuronal migration *via* the multipolar-to-bipolar transition of migrating neurons (Ohtaka-Maruyama et al., 2018). The subplate layer in primates is thicker than in rodents during embryonic brain development, suggesting that the subplate layer may play a distinguishing role in human-specific brain structure and neural circuits. Between GW9 and GW12, neurons generated in the VZ migrate toward the pial surface. Later born neurons pass these newly born neurons in an inside-out manner leading to the development of a mammalian-specific six-layered laminar structure. In human brain development, the formation of such a characteristic layered structure is thought to be involved in the expansion of the brain size and acquisition of higher functions (Figure 1).

After embryonic neurogenesis, RGs generate glial cells such as astrocytes and oligodendrocytes. Astrocytes play roles in the blood-brain barrier (BBB), regulation of synaptogenesis and synaptic activity, and interactions with oligodendrocytes and microglia (Khakh and Deneen, 2019; Santello et al., 2019). Oligodendrocytes form myelin sheaths, which increase the conduction velocity of action potentials by saltatory conduction, and play a role in the metabolism of neurons and astrocytes. When developing, oligodendrocyte progenitor cells (OPCs) differentiate into immature, mature, or myelinated oligodendrocytes (Fields, 2015). Each of these glial cells expressed different cell markers during differentiation. In recent years, single-cell transcriptome studies have revealed the vast diversity of glial cells that play roles in the development of the brain (Darmanis et al., 2015; Polioudakis et al., 2019; Fan et al., 2020; Huang et al., 2020). However, the mechanisms underlying oligodendrogenesis and white matter expansion in human remained largely unknown until recently. Remarkably, Huang et al. (2020) have reported that in human, oRGs produce EGFR-expressing pre-OPC in SVZ, then pre-OPC proliferates itself to increase the numbers of mitotic OPC in SVZ. They also reported that pre-OPC also proliferates and differentiates into early- and late-OPC in SVZ and SP, respectively (Huang et al., 2020). These findings provide the novel insight into the mechanism of oligodendrogenesis and white matter expansion in human brain development.

## Molecular Evolution for Human Brain

Functional changes in genes are a critical factor in the evolution of the human brain. Here, we discuss the relationship between the molecular evolution of the gene and human brain evolution and development, mainly focusing on ASD-associated genes.

*FOXP2, forkhead box P2* is one of the most well-known genes associated with human brain evolution and ASD. *FOXP2* belongs to the forkhead gene family of transcription factors. *FOXP2* was first identified in the KE family with speech and language phenotypes (Lai et al., 2001). Mutations of *FOXP2* in patients with speech and language have been linked to complete and complex orofacial sequential movements required for normal speech (Lai et al., 2001; Marcus and Fisher, 2003; Vargha-Khadem et al., 2005; Bacon and Rappold, 2012; Usui et al., 2017). Additionally, *FOXP2* mutations have been identified in patients with ASD (Gong et al., 2004; Li et al., 2005; Guo et al., 2019; Satterstrom et al., 2020; Wang et al., 2020). *FOXP2* is highly conserved among mammals, but two novel amino acids, T303N and N325S, arose in the protein sequence when the common ancestor of humans and chimpanzees diverged (Enard et al., 2002; Usui et al., 2014). These human-specific amino acid changes in FOXP2 have also been confirmed in Neanderthals (Krause et al., 2007; Green et al., 2010) and Denisovans (Reich et al., 2010), which further suggests that these two alterations may contribute to an acceleration in the molecular evolution of *FOXP2* functions (Enard et al., 2009; Konopka et al., 2009; Somel et al., 2013; Usui et al., 2014). Studies of *FOXP2* can provide novel insights into the molecular mechanisms underlying human brain evolution and communication.

*CLOCK* is a transcriptional factor that acts as a master regulator in controlling circadian rhythms (Takahashi, 2017). A 4q12 copy number variation that includes *CLOCK* has been linked to ASD (Sarachana et al., 2010; Griswold et al., 2012). In addition, a comparative genomics study of human and other primate brains revealed an increased expression of *CLOCK* only in the human prefrontal cortex (PFC), but not in chimpanzees and macaques (Konopka et al., 2012). Weighted gene co-expression network analysis (WGCNA) have identified *CLOCK* as a hub gene involved in cognitive disorder genes rather than in known circadian genes (Konopka et al., 2012). We further demonstrated in a previous study that *CLOCK* regulates human cortical neuronal migration as well as gene networks implicated in cognitive disorders, such as ASD and ID (Fontenot et al., 2017).

Mutations in the *ELAVL2* gene have also been identified in patients with ASD (Iossifov et al., 2014). *ELAVL2* is an RNAbp-regulating transcriptional and splicing network (Lenzken et al., 2014; Berto et al., 2016). RNA splicing plays a key role in brain development and ASD pathology (Grabowski, 2011; Voineagu et al., 2011; Irimia et al., 2014; Norris et al., 2014; Raj et al., 2014; Raj and Blencowe, 2015; Xiong et al., 2015; Bryant and Yazdani, 2016; Iijima et al., 2016). ELAVL2 is strongly co-expressed with several RNAbps, such as FMRP and RBFOX1, in human brain development (Berto et al., 2016). Additionally, *ELAVL2* targets are involved in neuronal development, synaptic function, and neurodegenerative disorders, such as ASD (Berto et al., 2016). Therefore, RNAbps such as *ELAVL2* may affect human brain evolution and development *via* RNA splicing, and mutations or abnormalities in this gene could be a predictive factor of NDDs.

Previous studies on molecular evolution have shown that changes in the nature and function of genes, like those mentioned above, in the evolution of the human brain are likely related to the evolution of higher cognitive functions and the associated risk of brain diseases such as NDDs.

## Roles of Oligodendrocyte in Human Brain Function

Human brain evolution was previously characterized by an increase in the number of neurons present over evolutionary time (Gabi et al., 2016; Preuss, 2017); however, the number of glia in the human brain, not the number of neurons explicitly, has increased dramatically, and the ratio of neurons to glia is higher in the human brain than in that of rodents and other primates (Sherwood et al., 2006). The human brain undergoes a volumetric expansion of white matter (Donahue et al., 2018; Rilling and van den Heuvel, 2018), and approximately 75% of non-neurons in the human cortex are oligodendrocytes (Pelvig et al., 2008; Herculano-Houzel, 2014). Oligodendrocytes wrap around neuronal axons and form a sheath of insulating myelin to accelerate the conduction of action potentials (Fields, 2015; de Faria et al., 2021). In recent years, it has been reported that oligodendrocytes may also play a role in the metabolism of lactic acid in the brain, and in the supplying of nutrients to nerve cells and astrocytes (Lee et al., 2012), all of which support the development of higher cognitive function. In fact, previous studies have specifically singled out oligodendrocyte dysfunction in both schizophrenia and major depressive disorder (MDD) (Miyata et al., 2015). Other studies have further demonstrated the involvement of oligodendrocytes in human cognitive function (Voineskos et al., 2013; Fields et al., 2014), suggesting that oligodendrocytes likely has essential functions in human brain evolution.

To test this idea, we previously carried out a comparative genomic study using postmortem brains of human and other primates, and the results suggested that oligodendrocytes may contribute to human brain evolution (Berto et al., 2019), which supports the findings of other previous studies. A human-specific down-regulated oligodendrocyte module is enriched for genes in a module dysregulated in ASD, schizophrenia, and bipolar disorder (BD), and is linked with transcription and methylation. In contrast, a human-specific upregulated oligodendrocyte module is enriched for genes in a module dysregulated in schizophrenia and linked with splicing, such as RNA metabolism and RNA processing. Furthermore, we demonstrated that human-specific genes are enriched for cognitive disease risk variants, such as ADHD, schizophrenia, BD, and MDD (Berto et al., 2019).

Recent studies have focused on the relationship between oligodendrocytes and these disorders. For example, age-related differences in white matter diffusion have been reported in the uncinate fasciculus, corticospinal tract, inferior longitudinal fasciculus, inferior fronto-occipital fasciculus, anterior thalamic radiation, superior longitudinal fasciculus, and forceps major of ASD (Thompson et al., 2020). Further studies on the severity of ASD and white matter development in early childhood have reported that development with ASD results in lower white matter development than in neurotypical development (Andrews et al., 2021). Furthermore, lipids are the main constituents of myelin; however, dyslipidemia has been reported in children with ASD (Usui et al., 2020). In a recent study using a mouse model, we reported a reduction in the myelinated area in the neocortex of the ASD-associated gene *Zbtb16* knockout mice (Usui et al., 2021). In addition, previous studies have reported similar abnormalities in the white matter of patients with ADHD (Wu et al., 2017; Bessette and Stevens, 2019). Large white matter tracts that form myelinated fibers are considerably smaller in the brains of patients with ADHD (Liston et al., 2011; van Ewijk et al., 2012). NOS1, encoding nitric oxide synthase 1, has been identified as a candidate gene for ADHD (Hawi et al., 2015). NOS1 promotes the growth and arborization of oligodendrocytes (Garthwaite et al., 2015), suggesting that myelination defects might contribute to the presentation of ADHD symptoms. Moreover, it has been reported that the alterations in white matter and oligodendrocyte-related genes regulation are associated with other disorders such as schizophrenia, BD, and MDD (Barley et al., 2009; Haroutunian et al., 2014; Srivastava et al., 2016; Tonnesen et al., 2018).

**FIGURE 2:**
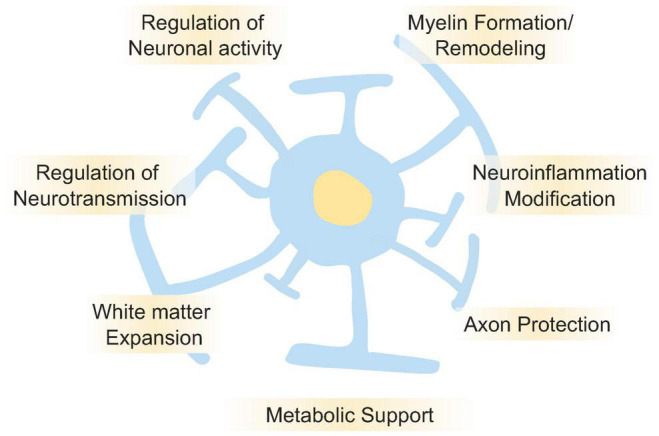
Roles of oligodendrocyte in the brain functions. Oligodendrocyte plays essential roles for regulation and maintenance of brain functions. It is well known that oligodendrocyte regulates neuronal activities by myelin formation and its remodeling. It also regulates neurotransmission, neuroinflammation, axon protection, and metabolic supports. Oligodendrocytes are one of the major cell types that make up white matter, and in the human brain, the expansion of white matter is thought to have contributed to higher brain functions. Disruption of those oligodendrocyte’s functions is also thought to cause NDDs, psychiatric disorders, and cognitive dysfunction.

Together, the findings of these studies suggest that evolutionary trajectories in oligodendrocytes are at risk of association with cognitive diseases as well as NDDs; regardless, oligodendrocytes play a role in human brain evolution and the acquisition of higher functions in the brain (Figure 2).

## Discussion

In this review, we have taken a multifaceted approach to evaluating the relationship between human brain evolution and NDDs, focusing on several factors such as development, molecular mechanisms, and comparative genomes. It has been suggested that molecular evolution at the gene and protein levels in humans as well as RNA splicing control mechanisms play a role in human brain evolution. Moreover, it is necessary to clarify in future research that oligodendrocytes accelerate brain evolution. Since oligodendrocytes play a human-specific role in metabolism and RNA splicing in the brain, further studies should evaluate the regulation of larger-scale energy metabolism in the evolved human brain and the diversity of various gene regulation mechanisms. In addition, most of the studies in this review are only focused on genetic and molecular mechanisms of evolution, but with little to discuss about the complex interplay of environmental factors that can also affect neurodevelopment. Therefore, future studies could try to integrate environmental factors along with genetic factors to better contextualize the evolution of the human brain and the pathophysiology of NDDs. In conclusion, this review synthesizes recent findings that suggest a role of evolutionary mechanisms in brain development and in the increased risk of NDDs.

## Author Contributions

KI: writing - original draft. MD: writing - original draft, and visualization. NU: conceptualization, writing - original draft, writing - review and editing, project administration, and funding acquisition. SS: writing - review and editing, supervision. All authors contributed to the article and approved the submitted version.

## Conflict of Interest

The authors declare that the research was conducted in the absence of any commercial or financial relationships that could be construed as a potential conflict of interest.

## Publisher’s Note

All claims expressed in this article are solely those of the authors and do not necessarily represent those of their affiliated organizations, or those of the publisher, the editors and the reviewers. Any product that may be evaluated in this article, or claim that may be made by its manufacturer, is not guaranteed or endorsed by the publisher.
